# T-Cells Subsets in Castleman Disease: Analysis of 28 Cases Including Unicentric, Multicentric and HHV8-Related Clinical Forms

**DOI:** 10.3390/ijms24097813

**Published:** 2023-04-25

**Authors:** Sara Fraticelli, Marco Lucioni, Giuseppe Neri, Deborah Marchiori, Caterina Cristinelli, Michele Merli, Rodolfo Monaco, Tiziana Borra, Antonio Lazzaro, Silvia Uccella, Luca Arcaini, Marco Paulli

**Affiliations:** 1 Department of Molecular Medicine, University of Pavia, 27100 Pavia, Italy; sara.fraticelli@unipv.it (S.F.); marco.lucioni@unipv.it (M.L.); giuseppe.neri01@universitadipavia.it (G.N.); caterina.cristinelli01@universitadipavia.it (C.C.);; 2 Pathology Unit, Fondazione IRCCS Policlinico S. Matteo, 27100 Pavia, Italy; 3 Pathology Unit, Department of Medicine and Surgery, University of Insubria, 21100 Varese, Italy; 4 Division of Hematology, Ospedale di Circolo e Fondazione Macchi, 21100 Varese, Italy; michele.merli@asst-settelaghi.it; 5 Pathology Unit, Ospedale Guglielmo da Saliceto, 29121 Piacenza, Italy; 6 Department of Pathology, Azienda Ospedaliera SS Antonio e Biagio e Cesare Arrigo, 15121 Alessandria, Italy; tiziana.borra@ospedale.al.it; 7 Division of Hematology and Bone Marrow Transplant Center, Ospedale Guglielmo da Saliceto, 29121 Piacenza, Italy; 8 Department of Biomedical Sciences, Humanitas University, Pieve Emanuele, 20072 Milan, Italy; silvia.uccella@hunimed.eu; 9 Pathology Service, IRCCS Humanitas Research Hospital, Rozzano, 20089 Milan, Italy; 10 Division of Hematology, Fondazione IRCCS Policlinico S. Matteo, 27100 Pavia, Italy

**Keywords:** Castleman disease, T-cell, CD8, FOXP3, T-reg, immunophenotyping, UCD, iMCD, HHV8 + MCD, lymphoproliferative disorder

## Abstract

Castleman disease (CD) is a rare lymphoproliferative disorder that includes various clinico-pathological subtypes. According to clinical course, CD is divided into unicentric CD (UCD) and multicentric CD (MCD). MCD is further distinguished based on the etiological driver in herpes virus-8-related MCD (that can occur in the setting of HIV); in MCD associated with POEMS syndrome (polyneuropathy, organomegaly, endocrinopathy, monoclonal protein, and skin changes); and idiopathic MCD (iMCD). The latter can also be divided in iMCD-TAFRO (thrombocytopenia, anasarca, fever, myelofibrosis, organomegaly) and iMCD not otherwise specified. To date, CD pathogenesis is still uncertain, but CD may represent the histological and clinical result of heterogeneous pathomechanisms. Transcriptome investigations in CD lymph nodes have documented the expression and up-regulation of different cytokines; furthermore, few recent studies have shown alterations of different T-cell subsets in CD patients, suggesting a possible role of the nodal microenvironment in CD development. On this basis, our study aimed to investigate the distribution of T-cell subsets in the clinico-pathological spectrum of CD. We evaluated the CD4/CD8 ratio and the number of T-regulatory (T-reg) FOXP3+ cells in 28 CD cases. In total, 32% of cases showed a decreased CD4/CD8 ratio due to increased CD8+ T-cells, including both UCD, iMCD, and HHV8+ MCD cases. The T-reg subset analysis revealed a statistically significant (*p* < 0.0001) lower mean number of FOXP3+ T-reg cells in CD cases when compared with non-specific reactive lymph nodes. We did not find statistically significant differences in T-reg numbers between the different CD subtypes. These findings may suggest that alterations in T-cell subpopulations that can lead to disruption of immune system control may contribute to the numerous changes in different cellular compartments that characterize CD.

## 1. Introduction

Castleman disease (CD) identifies a group of heterogeneous lymphoproliferative disorders included by the recent WHO classification among the tumor-like lesions with B-cell predominance [1].

Clinically, CD can present unicentric or multicentric lymphadenopathies. Unicentric CD (UCD) patients usually have no or mild symptoms, whereas multicentric CD (MCD) patients may present symptoms of severe inflammation, frequent cytopenia, and organ dysfunction [2,3,4].

Based on the etiological driver, MCD can be distinguished into herpes virus-8 (HHV-8)-related MCD with or without HIV coinfection (HIV−/+); MCD associated with polyneuropathy, organomegaly, endocrinopathy, monoclonal protein, and skin changes (POEMS); and idiopathic MCD (iMCD). The iMCD is furtherly distinguished into iMCD not otherwise specified (NOS), often associated with thrombocytosis and hypergammaglobulinemia, and iMCD presenting with thrombocytopenia, anasarca, fever, myelofibrosis, and organomegaly (TAFRO syndrome) [5].

Histopathologic descriptions of CD include a hyaline-vascular subtype (HV) clinically presenting as unicentric disease, a hypervascular subtype (HyperV) presenting as multicentric disease (iMCD) often in the setting of iMCD-TAFRO, a plasma cell (or plasmacytic, PC) subtype, and mixed forms that are in between [2]. In the HV subtype, the germinal centers (GC) have regressive/atrophic features, penetrating hyalinized vessels and an expanded mantle zone resulting in the so-called onion skin appearance. In addition, follicular dendritic cells (FDCs) are prominent, and sometimes dysplastic [6,7,8,9]. Although the HyperV subtype exhibits some histological similarities with the HV subtype, such as marked vascular proliferation and atrophic germinal centers, they are clearly distinct in terms of clinical presentation (unicentric versus multicentric disease); in addition, HyperV-type MCD often shows only slightly enlarged lymph nodes, whereas HV-type UCD is usually characterized by large localized masses [10]. Lastly, in the HyperV subtype, the nodal architecture is less distorted and FDCs dysplasia is less frequently observed [5].The PC-type shows reactive GCs and profuse plasmacytosis [11]; nodal architecture, and FDCs meshwork being usually preserved. In HHV8 + CD, hyperplastic follicles with normal appearing GCs are associated with a variable number of plasmocytic-plasmoblastic cells staining for LANA-1 [9,11,12].

Despite the different forms of CD often sharing histological features, the current hypothesis is that CD may represent the clinico-pathologic result of heterogeneous pathomechanisms [13]. Transcriptome investigations in CD have documented the expression and up-regulation of FDCs markers, angiogenetic factors, extracellular matrix remodeling factors, complement components, and markers for GC activation [14,15]. These findings point out that the nodal microenvironment, including its immune cellular components, plays a crucial pathogenetic role in CD.

Fajgenbaum et al. reported a CD8+ T-cells increase in the peripheral blood of patients with iMCD [16] and iMCD-TAFRO during disease flares [17]. Another study observed a decrease of T-follicular helper (Tfh) cells in lymph nodes from iMCD patients in comparison with non-specific reactive lymphadenopathies. The reduction was more pronounced in the iMCD-TAFRO patients, suggesting a pathogenetic role for this T-cell subset [18].

Nevertheless, only limited data are available concerning the distribution of the various T-cell subsets, including the T-regulatory (T-reg) lymphocytes in the different CD subtypes. T-reg lymphocytes are essential for both suppressing the immune response and maintaining self-tolerance and they have been advocated to play a key role in autoimmune and neoplastic disorders, facilitating tumor immune escape [19,20].

The aim of this study is to provide additional data about the distribution of T-cell subsets, including the FOXP3+ T-reg lymphocytes, in the clinico-pathological spectrum of CD.

## 2. Results

### 2.1. Clinical Features

The present series consists of 28 CD cases, respectively, 13 UCD, 9 iMCD, 5 MCD HHV8+/HIV−, and 1 MCD HHV8+/HIV+. The major clinical findings are summarized in Table 1.

#### 2.1.1. UCD

The UCD subtype included 13 patients, with a median age at diagnosis of 52 years (mean 44; range 6–67) and a female prevalence (9/13, 69%). Only one patient reported B-symptoms (weight loss) at diagnosis; no one presented organomegaly, cherry hemangiomas, or interstitial pneumonia, but two had a minimal fluid collection (one pleural effusion and one Douglas pouch effusion).

Tests for HHV8 (respectively, immunostainings and/or circulating DNA), HIV, HCV, and HBV were negative in all tested cases. One patient had a previous CMV infection (documented by presence of IgG antibodies), while two had previous EBV infection.

AA Amyloidosis was described in two patients (2/13, 15%).

Serum IL6 was increased in two out of five tested patients (range 0.3–109.1); a monoclonal component was detected in two cases.

In total, ten patients had surgical excision only, and one patient received corticosteroids after CD diagnosis on a lymph node biopsy. At last follow-up (FU), 11 patients (84.6%) were alive without disease, while 2 were lost at follow-up (LFU).

#### 2.1.2. MCD

##### iMCD

The iMCD subtype included nine patients with a median age at diagnosis of 57 years (mean 56, range 40–65) and a male prevalence (6/9, 67%). No patients had clinical symptoms related to POEMS or TAFRO syndrome, therefore, they were all classified as iMCD NOS. We did not observe any case showing clinico-pathologic features consistent with the so-called iMCD-IPL (idiopathic plasmacytic lymphadenopathy) variant [10,21]. No patients had cherry hemangiomas. At diagnosis, four out of seven patients had B-symptoms and five out of eight had organomegaly. HCV and HIV tests were negative in all tested cases; in four out of six cases a previous infection by CMV (IgG positive antibodies) was documented; a previous EBV infection was detected in four out of six patients.

There were two patients that had a diagnosis of autoimmune disease prior to the CD diagnosis (systemic sclerosis with limited cutaneous involvement, erythema nodosum). No IgG4-related disease was observed. There were two cases of peripheral neuropathy that were documented, and two patients that developed a lymphoproliferative disorder during FU, respectively, multiple myeloma and bone plasmocytoma (about one year after CD diagnosis in both cases).

The serum IL6 was increased in three out of five tested patients (range 1.59–95 pg/mL); a monoclonal component was detected in all seven tested patients.

Treatment programs were available in five out of nine iMCD cases. The patients were treated with Siltuximab (*N* = 3), Cyclophosphamide and steroids (*N* = 1), or cyclophosphamide, vincristine, prednisone (CVP) (*N* = 1); two patients underwent second line therapy with chemoimmunotherapy.

At last FU five patients (5/9, 56%) were still alive, two (2/9, 22%) were dead for other causes (DOC), while two (2/9, 22%) were LFU.

##### HHV8+/HIV− MCD

In total, five patients were analyzed, all males and with a median age at diagnosis of 75 years (mean 69, range 35–83). One case had a previous history of HIV− Kaposi sarcoma. B-symptoms and hepatosplenomegaly at diagnosis were observed in three cases. Hemophagocytic lymphohistiocytosis was reported in two patients.

HCV tests were negative in all tested cases (0/5) but one patient had immunity for HBV. One patient had an active CMV infection, one a viral reactivation, and another one a previous infection.

The serum IL-6 value was available in a single case only, and it was increased (67.76 pg/mL).

Treatment schedules were available in two cases: one underwent rituximab single agent therapy, the other received liposomal doxorubicin as first line therapy and paclitaxel and etoposide as second and third line, respectively, because of concurrent Kaposi sarcoma.

At last FU, one patient (1/5, 20%) was DOC, two (2/5, 40%) were dead for complications that were disease-related (i.e., hemophagocytic lymphohistiocytosis), while two (2/5, 40%) were LFU.

##### HHV8+/HIV+ MCD

A 54-year-old patient presented at diagnosis with hepatosplenomegaly and B-symptoms. He had a previous history of HIV+ Kaposi sarcoma. HCV and HBV tests were negative, but he had previous CMV and EBV infections.

Serum IL6 was increased (44.4 pg/mL) and a monoclonal component was detected.

The treatment regimen included liposomal doxorubicin and rituximab; at last FU the patient was still alive.

### 2.2. Histopathological Features

At histopathological evaluation, 11/13 UCD patients showed an HV histology, whereas the remaining two had a mixed one; MCD patients showed a PC histology in 3/15 cases, a mixed one in 10/15 and HyperV in 2/15.

In one patient (iMCD with mixed histology), the CD changes were detected both in the lymph nodes and in the lymphoid cellular infiltrate involving the subcutis of the left thigh; bone marrow CD localization was found in a single case (HHV8+/HIV− MCD with mixed-type histology).

Follicle B centers with hyperplastic/dysplastic follicular dendritic cells were found in 5/28 (18%) cases (three HV UCD, one mixed-type MCD, and one PC-type MCD).

In most cases (26/28, 2 cases could not be evaluated due to technical issues) the plasma cell population showed polytypic Ig light chains expression by immunohistochemistry; 3/28 had an IgG4/IgG > 40% and IgG4 > 10/HPF, but did not meet the diagnostic criteria for IgG4-related disease [22,23,24]

EBV, tested by in situ hybridization (ISH) technique, was negative in all cases (28/28).

### 2.3. T-Cell Subset Analysis

#### 2.3.1. CD4/CD8 Analysis

**Figure 1:**
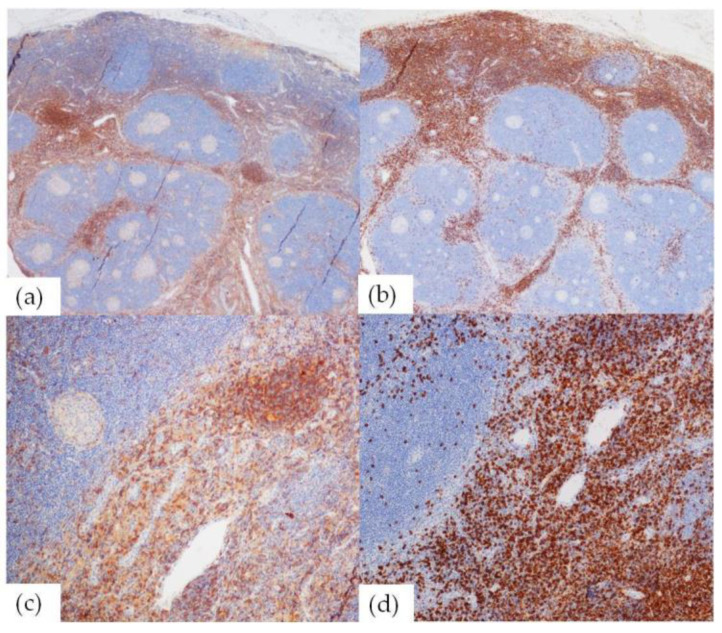
Nodal immunostainings show a CD4/CD8 ratio in favor of CD8+ T-cell populations, noticeable at both low (**a**) CD4+ T-cells (20×); (**b**) CD8+ T-cells (20×), and higher magnification (**c**) CD4+ T-cells (100×); (**d**) CD8+ T-cells (100×).

The CD4/CD8 ratio was decreased in nine cases. One case (HHV8+HIV+MCD mixed) showed a predominance of CD8+ T-cells, four cases showed a similar amount of CD4+ and CD8+ T-cells (three UCD HV-type, one UCD mixed-type), four cases had still a predominance of CD4+ T-cells but with a significant increase in the CD8+ population (one UCD HV-type, one UCD mixed-type, one iMCD PC-type, and one HHV8+ MCD mixed-type). (Figure 1)

**Figure 2:**
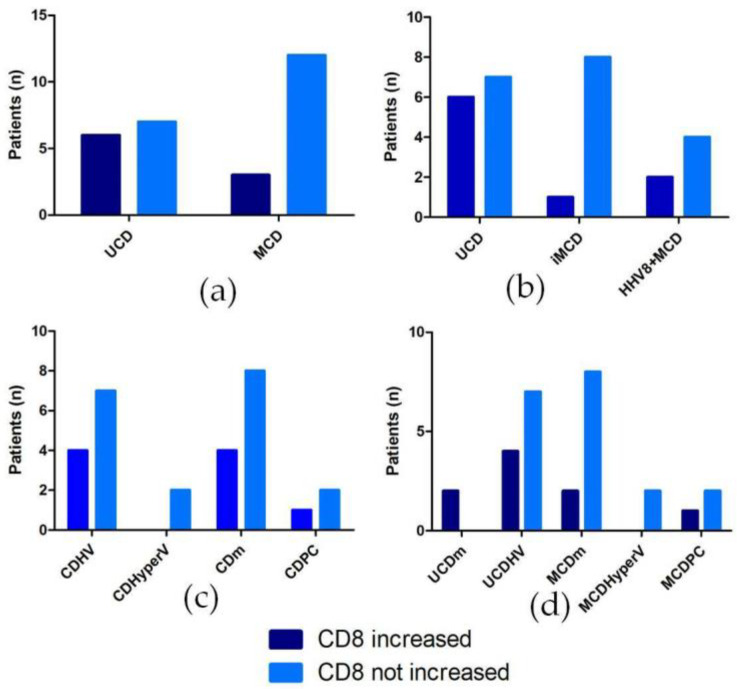
Evaluation of CD4/CD8 ratio in different CD subtypes. (**a**) Evaluation of decreased CD4/CD8 ratio vs. normal CD4/CD8 ratio in UCD and MCD cases; (**b**) Evaluation of decreased CD4/CD8 ratio vs. normal CD4/CD8 in UCD vs. iMCD vs. HHV8+ MCD; (**c**) Evaluation of decreased CD4/CD8 ratio vs. normal CD4/CD8 in the various histological subtypes; (**d**) Evaluation of decreased CD4/CD8 ratio versus normal CD4/CD8 in the various clinico-histological subtypes. Dark blue bars indicate cases with a decreased CD4/CD8 ratio (increased quantity of CD8+ cells), light blue bars indicate cases with a normal CD4/CD8 ratio. Abbreviations: UCD, unicentric Castleman disease; MCD, multicentric Castleman disease; iMCD, idiopathic multicentric Castleman disease; UCDm, unicentric Castleman disease mixed-type; UCDHV, unicentric Castleman disease hyaline-vascular-type; MCDm, multicentric Castleman disease mixed-type; MCDHyperV, multicentric Castleman disease hypervascular-type; MCDPC, multicentric Castleman disease plasma cell-type.

No statistically significant differences in terms of CD4/CD8 ratio changes were found when comparing different clinical (UCD vs. MCD (as a whole), *p* = 0.2275; UCD vs. iMCD vs. HHV8+MCD, *p* = 0.2232); histological (CD HV vs. CD HyperV vs. CD mixed vs. CD PC, *p* = 0.7899); and clinico-pathological subtypes of CD (HV UCD vs. mixed UCD vs. HyperVMCD vs. mixed MCD vs. PC MCD, *p* = 0.2039) (Figure 2).

Architectural assessment of T-cell subpopulations revealed that both CD4+ and CD8+ T-cells were distributed in their specific nodal paracortical/interfollicular compartments; CD4+ T-cells being more numerous around the germinal centers and CD8+ lymphocytes beyond.

#### 2.3.2. T-reg Analysis

Immunohistochemical staining for FOXP3+ T-reg was carried out on 28 CD and 24 control samples (reactive lymph nodes). The number of FOXP3+ T-cells in CD-affected lymph nodes was significantly (*p* < 0.0001) lower (mean 23.18 ± 20.18) in comparison to the number in the control samples (mean 58.63 ± 28.04). In the UCD, the numbers of FOXP3+ T-reg (mean 26.54 ± 26.87) were compared, respectively, to the numbers of T-reg in MCD cases as a whole (mean 20.27 ± 12.11), in iMCD (mean 17.33 ± 12.04), and in HHV8+ MCD (mean 24.67 ± 11.83). However, the statistical analysis of T-reg distribution in these different CD clinical subtypes did not reveal any statistically significant differences (*p* = 0.9083; *p* = 0.5965) among the different CD forms.

Similarly, we compared the FOXP3+ T-reg numbers in the different CD histologic subtypes, respectively, HV (mean 27 ± 29.07), HyperV (mean 28.5 ± 6.36), PC (mean 22.33 ± 4.51), and mixed (mean 19 ± 13.59) but no statistically significant differences (*p* = 0.7824) emerged. Subsequently, we compared the number of FOXP3+T-reg with respect to the different clinico-pathological CD subtypes, including, respectively, UCD HV (mean 27 ± 29.07), UCD mixed (mean 24 ± 14.14), MCD HyperV (mean 28.5 ± 6.364), MCD mixed (mean 18 ± 14.03), and MCD PC (mean 22.33 ± 4.509); in this analysis, no statistically significant differences (*p* = 0.8205) were found.

**Figure 3:**
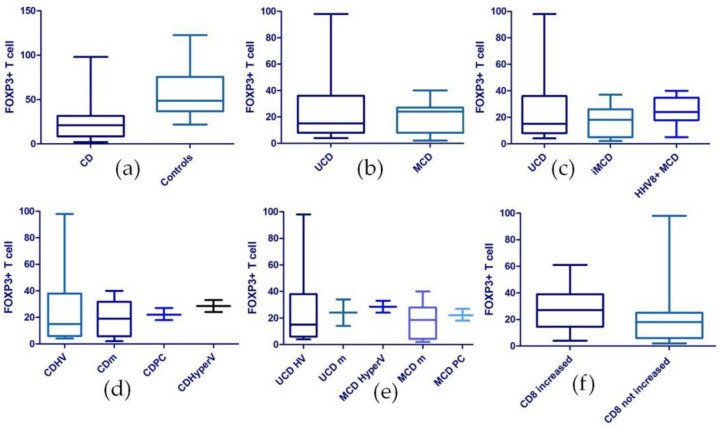
FOXP3 analysis. (**a**) FOXP3 analysis in CD patients versus controls shows a statistically significant (*p* < 0.0001) difference between the 2 groups; comparison of FOXP3+ cells instead did not show statistically significant differences between the various CD clinical subtypes: (**b**) UCD vs. MCD; (**c**) UCD vs. iMCD vs. HHV8+ MCD. Likewise, statistically significant differences were not found comparing (**d**) the various pathological subtypes or (**e**) the different clinico-pathological subtypes nor (**f**) comparing cases with an increased CD8+population vs. cases without. Abbreviations: CD, Castleman disease; UCD, unicentric Castleman disease; MCD, multicentric Castleman disease; iMCD, idiopathic multicentric Castleman disease; HV, hyaline-vascular; HyperV, hypervascular; m, mixed-type; PC, plasma-cell-type.

The comparison between the number of FOXP3+ T-reg in cases with an increase in the CD8+ T-cell population (mean 28.56 ± 17.04) and cases without CD8+ T-cell subset increase (mean 20.63 ± 21.45) revealed no statistically significant difference (*p* = 0.0991) (Figure 3)

## 3. Discussion

Several studies contributed to highlight the major biological features of CD, mainly including the central pathogenetic role of IL-6 and other inflammatory cytokines [2,11,25,26,27]. Furthermore, data accumulated pointing out the crucial role of local (nodal) microenvironment among the major sources of hypercytokinemia [14,15]. In this respect, it seems reasonable that the interplay between the T-cell populations and other components of the local (nodal) microenvironment might play a crucial role for defining the inflammatory milieu and the various modalities of the immune response.

Nevertheless, only a few previous studies have focused on the nodal cellular composition and distribution of accompanying T-cell subsets in the different CD subtypes [17,18].

The aim of this study is to contribute to elucidate the immunophenotypic features and distribution of the T cellular background in nodal biopsies within the different subtypes of CD.

The CD4+/CD8+ T-cells ratio assessment documented an increase in CD8+ T-cells in 32% of CD patients. Such an increase occurred irrespective of the clinico-pathological subtype of CD but it was more frequently associated with the UCD (6/13 cases, 46%, 4 of them exhibiting HV histology and 2 mixed histology).

In the others CD subtypes, the CD8+ T-lymphocytes nodal tissue levels were increased in 1/9 (11%) iMCD, 1/5 (20%) HHV8+ HIV− MCD, and in the single case of HHV8+ HIV+ MCD. As to the histopathologic features of the MCD cases with altered CD4/CD8 ratio, the single case of iMCD had a PC histology, whereas both HHV8+ cases showed a mixed histology.

Fajenbaum et al. reported an increased level of CD8+ T-cells in the peripheral blood of patients with iMCD [16] and iMCD-TAFRO, during disease flares by means of flow cytometry analysis [17]. Furthermore, in the iMCD-TAFRO study, the proportions of CD4+ and CD8+ T-cells were also assessed in lymph node tissue by flow cytometry, showing an increased proportion of CD8+ T-cells in both analyzed cases [17].

Our data document that increased CD8+ T-cell levels may occur also in other CD forms including UCD and HHV8+ MCD, suggesting that imbalances in the CD4/CD8 ratio may be shared by different CD subtypes.

Studies about the neoplastic microenvironment in murine models showed that IL-6 might mobilize a T-cell antineoplastic immune response, promoting differentiation of naive CD8+ T-cells in specific CD8+ effectors [28,29,30]. On such findings, we might suppose that increased IL-6 levels in CD could similarly favor the expansion of CD8+ T-cell subpopulations. The limited data on IL-6 values in our series prevent any significative conclusions, nevertheless the three patients with a decreased CD4/CD8 ratio with availability of serum IL-6 values also showed serum IL-6 values higher than normal.

As at least a subset of CD might be associated with the disruption of immune system control, we tried to assess the role of a peculiar subset of regulatory T-cells, known as T-reg that can be identified by means of specific immunophenotypic markers. T-reg lymphocytes are a subpopulation of CD4+ T-lymphocytes, characterized by CD25 and FOXP3 expression, that are involved in immune response suppression and self-tolerance maintenance. They exert their function in maintaining immune homeostasis by controlling immune responses and through mutual regulation between T-reg and T-effector, thus playing a relevant role in both preventing autoimmunity and facilitating tumor immune escape [19,31]. Various studies have documented increased T-reg levels in both solid and lymphoid malignancies, probably contributing to tumor immune evasion by suppressing the anti-tumor T-effector response [20,32,33,34,35]; adversely reduced T-reg levels and/or their functional impairment can result in T-effector responses against self-antigens leading to autoimmune diseases [36].

On this basis, we assessed the number of FOXP3+ T-reg cells in our series as well as in controls (non-specific reactive lymph nodes). Immunostainings revealed a lower mean number of FOXP3+ T-reg cells in CD cases when compared with controls (23.18 in CD vs. 58.63 in reactive lymph nodes). This difference in FOXP3+ T-reg cells nodal tissue levels was statistically significant by means of a Mann–Whitney test (*p* < 0.0001). In contrast, no statistically significant difference in the number of T-reg lymphocytes was found among the different CD clinico-pathological subsets. We also found no statistically significant correlation between the FOXP3+ T-reg count and the CD4/CD8 ratio. The decreased number of FOXP3+ T-reg lymphocytes we observed suggests that pathogenetic mechanisms similar to those that can lead to autoimmune diseases might also be involved in CD. Conversely, a few studies documented an increased level of intra-tumoral T-reg cells in patients with B-cell lymphomas, leading, in some cases, to the inhibition of intralesional CD8+ T-cells [20,32]. Based on these findings, we may hypothesize that CD develops through different pathways than most B-cell lymphomas.

In conclusion, this study has documented that in all the different clinico-pathological subtypes of CD, changes may occur in the composition of nodal CD T-cell background, particularly including a frequent decrease of the CD4/CD8 ratio and a reduction in FOXP3+ T-reg cells. However, the limited number of analyzed cases prevents us from making any definitive conclusions. Further studies, particularly based on transcriptomic analysis, are needed to better elucidate the functional relationships between the T-cellular subsets and other components of the local microenvironment in CD.

## 4. Materials and Methods

### 4.1. Patients Selection

Patients who had a previous diagnosis of CD in between 2000 and 2022 were collected from the databases of IRCCS San Matteo Hospital Foundation of Pavia, Hospital of Varese and of Guglielmo da Saliceto Hospital of Piacenza.

A group of experienced hematopathologists (MP, SU, ML) reviewed all diagnostic slides (i.e., H&E and immunohistochemical stains).

Diagnoses of CD were made according to MCD and UCD criteria [5,6], see Appendix A; diagnosis of HHV8+ CD was made when there was a positive testing for HHV8 by LANA-1 on lymph node tissue and/or a positive testing for circulating HHV8 DNA [2].

In total, 36 patients with a CD diagnosis were initially collected and following histological revision, 28 patients were, therefore, included in the study. We retrieved a dataset of epidemiological, pathological, and clinical information including: age, sex, diagnosis, presence of B-symptoms, presence of hepatomegaly, splenomegaly, bone marrow involvement, fluid effusions, skin alterations, autoimmune diseases, POEMS, TAFRO, lymphoproliferative disorders, other diseases; laboratory data (hemoglobin, LDH, creatinine, presence of a monoclonal component, serum IL-6), virological status (HHV8, HIV, HBV, HCV, EBV, CMV), bacterial infection (tuberculosis); and histopathological features, therapy, outcome and follow-up. At the end of follow-up, patients were defined as alive with/without disease, dead for complications that were disease-related, DOC, and LFU.

We obtained informed consent from all patients in accordance with local ethical guidelines and with the Helsinki Declaration of 1975.

### 4.2. Pathological Methods

Formalin-fixed, paraffin-embedded (FFPE) biopsies from all included cases were available for histopathological studies and stained with Hematoxylin and eosin (HE) and Giemsa.

Immunohistochemical analysis with antibodies against CD20, CD79a, BCL2, CD10, BCL6, CD138, CD3, CD5, κ, λ, IgG, IgG4, Mib1/Ki-67, CD21, CD23, Cyclin D1, CD68R/PGM1, CD34, HHV8/LANA-1 (Agilent/Dako, Santa Clara, CA, USA), were performed using the automated platform Dako Omnis Envision Flex.

All cases were tested for EBV presence by means of ISH, using Epstein–Barr virus (EBER) peptide nucleic acid (PNA) Probe linked with fluorescein for the detection of latent EBV infection on FFPE tissue sections.

### 4.3. T-Cell Subset Analysis

We used immunohistochemical analysis with antibodies against CD4 and CD8 T-cell subsets to evaluate the CD4/CD8 ratio in all the affected lymph nodes. The CD4/CD8 ratio was assessed in the paracortical areas using a semiquantitative approach. Adjacent sections were immunostained, respectively, with antibodies against CD4 and CD8. The CD4/CD8 ratio was assessed by 4 pathologists on digital photographs of representative fields.

An antibody against FOXP3 (eBioscience/Thermofisher, Waltham, MA, USA), a specific marker of T-reg population [31], was employed to analyze this T-cell subset.

To evaluate the number of FOXP3+ cells, 10 high power fields (HPF) for each case were selected from areas of more prominent aggregation, and the number of cells with nuclear positivity were counted under a light microscope. The mean number of FOXP3+ T-cells was calculated for each analyzed case.

For T-reg subset analysis, we collected 24 non-specific reactive lymph nodes from different sites (i.e., cervical, supraclavicular, axillary, abdominal, iliac, inguinal) to be used as a control group.

### 4.4. Statistical Analysis

The data were described with the mean and standard deviation if continuous and with counts and percentages if categorical; they were compared between groups with the Student’s *t* test/Mann–Whitney or the Fisher/χ^2^ test, respectively. A two-sided *p*-value < 0.05 was considered statistically significant.

## Figures and Tables

**Table 1:** Main clinical features.

Clinical and Laboratory Features	All Patients (*N* = 28)			
	UCD	iMCD	HHV8+ HIV− MCD	HHV8+ HIV+ MCD
B-symptoms *	1/10	4/7	3/4	1/1
Anemia (Hb < 13 g/dL M, <12 g/dL F)	1/10	2/7	4/4	1/1
Serum monoclonal component **	2/7	7/7	1/3	1/1
Hepatosplenomegaly	0/10	5/8	3/4	1/1
Fluid effusions	2/10	0/7	1/3	0/1
Increased creatininemia (>1.2 mg/dL)	0/9	0/7	3/4	0/1
Proteinuria (>15 mg/dL)	1/6	2/6	2/4	1/1
Interstitial lymphocytic pneumonia (LIP)	0/9	0/9	0/4	0/1
Peripheral neuropathy	0/13	2/9	0/4	0/1
LDH > UNL	4/10	4/6	1/4	1/1
IL-6 > UNL	2/5	3/5	1/1	1/1
HHV8 (circulating DNA and/or IHC) +	0/13	0/9	5/5	1/1
EBV serology (IgG: EBNA, VCA) +	3/4	4/6	2/2	1/1
EBV-DNA+	0/4	0/5	1/2	0/1
CMV serology (IgG) +	3/5	4/6	2/3	1/1
CMV-DNA+	0/5	0/6	2/3	0/1
HIV serology +	0/9	0/7	0/5	1/1
HCV serology +	0/9	0/7	0/5	0/1
HBV serology (anti-HBc antibodies) +	0/9	2/7	1/5	0/1
Quantiferon TB test +	0/5	0/4	0/2	0/1
Cherry hemangiomas	0/10	0/9	0/4	0/1
Secondary amyloidosis	2/13	0/9	0/5	0/1
Lymphoproliferative clonal disorder	0/13	2/9	0/4	0/1
Hemophagocytic lymphohistiocytosis	0/13	0/9	2/4	0/1
Solid tumor	1/13	2/9	2/4	1/1
Autoimmune disease ***	0/13	2/9	1/4	0/1
POEMS syndrome	-	0/9	0/4	0/1
TAFRO syndrome	-	0/9	0/4	0/1

* at least one between night sweats, fever, fatigue G > 2 CTCAE, weight loss. ** monoclonal spike detected on serum protein electrophoresis and/or an abnormal (positive) serum immunofixation *** other than systemic lupus erithematosus, rheumatoid arthritis, Adult Still’s disease, and autoimmune lymphoproliferative syndrome. Abbreviations: Hb, hemoglobin; LDH, lactate dehydrogenase; UNL, upper normal limit; IL-6, interleukin-6; HHV8, human herpes virus-8; IHC, immunohistochemistry; EBV, Epstein–Barr virus; IgG, Immunoglobulin G; EBNA, Epstein–Barr nuclear antigen; VCA, viral capsid antigen; CMV, cytomegalovirus; HIV, human immunodeficiency virus; HCV, Hepatitis C virus; HBV, Hepatitis B virus; HBc, Hepatitis B core antigen; TBC, tuberculosis.

**Figure A1:**
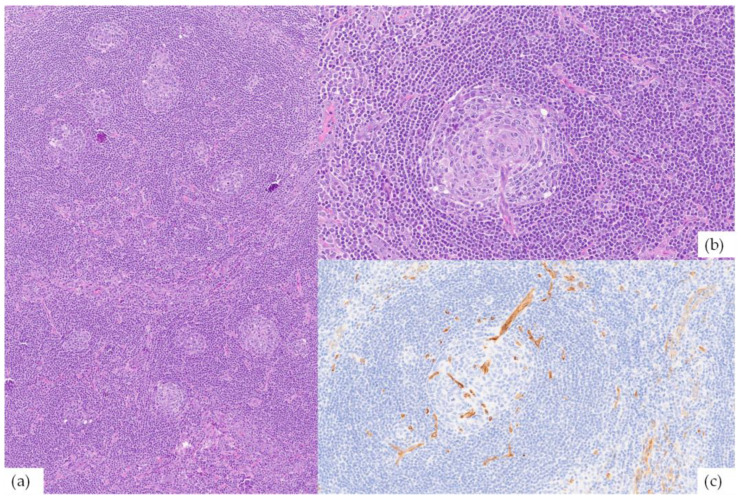
Hyaline vascular histology shows multiple regressed germinal centers within the same, expanded mantle zone (“twinning”) (**a**), hematoxylin and eosin, 100×, and increased vascularity with radially penetrating vessels (“lollipop”) (**b**), hematoxylin and eosin, 200×; (**c**), CD34 immunostaining, 200×.

**Figure A2:**
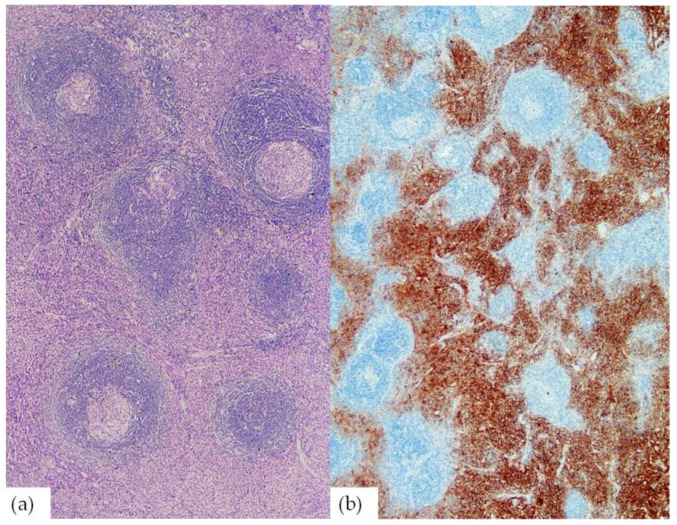
Plasma cell histology shows variably sized, mostly hyperplastic, germinal centers (**a**), hematoxylin and eosin 100×, and sheet-like plasmacytosis (**b**), CD138, 100×.

## Data Availability

The data presented in this study are available on request from the corresponding author.

## Appendix A. Histopathological Features

Histopathological features of lymph nodes with CD may range from the so-called hyaline vascular (HV, in UCD), hypervascular, (HyperV, in iMCD setting), to plasma cell or plasmacytic (PC) subtype. The HV subtype shows follicular hyperplasia with altered (atresic) germinal centers, usually depleted of B cells, expanded mantle zones composed of small lymphoid cells (“onion skin appearance”), and increased vascularity with hyalinized, pathological blood vessels radially penetrating the GC (“lollipop lesions”). FDCs may be hyperplastic and/or dysplastic. The HV subtype is also characterized by multiple germinal centers within the same mantle zone (“twinning”) and patent sinuses; instead, sheets of plasma cells are not seen. The HyperV subtype exhibits some histologic similarities with the HV subtype, such as marked vascular proliferation and atrophic germinal centers; however, in the former, the lymph node architecture is often less severely disrupted and FDCs dysplasia is less frequently observed. On the other hand, the key morphological feature of the PC subtype is the presence of sheets of plasma cells in the interfollicular zone, associated with variably sized GC (usually hyperplastic). PC cases also have occasional regressed GC and mild vascularity, the “onion skin” mantle zone and the penetrating blood vessels may still be seen in some cases. Cases which demonstrate intermediate histological features between the HV, HyperV, and PC subtypes are considered as “mixed” [5,6,11].

Every case was assessed according to the grading system established by Fajgelbaum et al. [5,6] (see Table A1).

Based on such criteria, we achieved the diagnosis of HV or HyperV CD in cases with prevalence of regressed germinal centers and increased vascularity (see Table A1 and Figure A1); in cases with sheet-like plasmacytosis and hyperplastic follicles, we made a diagnosis of PC CD (see Table A1 and Figure A2). Lastly, cases with features in between these two subtypes were categorized as mixed.

**Table A1:** Histological features UCD and iMCD according to grading system by Fajgelbaum et al. [5,6].

Case	UCD/iMCD	Histologic Subtype	Regressed GC	FDC Prominence	Vascularity	Hyperplastic GC	Plasmacytosis
1	UCD	HV	3	3	2	1	0
2	UCD	HV	3	1	2	0	0
4	UCD	mixed	2	1	1	2	2
5	UCD	HV	2	2	1	2	0
6	UCD	HV	2	2	1	1	0
7	iMCD	mixed	2	1	2	1	2
9	UCD	HV	2	2	2	1	0
10	UCD	HV	3	2	0	0	0
11	UCD	mixed	1	3	0	3	2
13	iMCD	plasma cell-type	2	2	1	1	3
14	iMCD	HyperV	2	2	1	1	0
16	iMCD	mixed	2	2	1	2	2
18	iMCD	mixed	3	2	1	1	2
19	UCD	HV	2	3	1	2	0
22	iMCD	mixed	3	2	2	1	2
24	iMCD	HyperV	3	0	1	0	1
25	iMCD	mixed	2	1	2	1	3
26	UCD	HV	2	2	1	0	0
28	UCD	HV	2	3	3	1	2
30	iMCD	plasma cell-type	1	2	2	1	3
32	UCD	HV	2	2	3	2	1
33	UCD	HV	2	1	1	0	0

Abbreviations: UCD, unicentric Castleman disease; MCD, multicentric Castleman disease; GC, germinal centers; FDC, follicular dendritic cell; iMCD, idiopathic multicentric Castleman disease; HV, hyaline-vascular-type; HyperV, hypervascular-type.
